# Case of Purpura Hœmorrhagica

**Published:** 1849-08

**Authors:** S. C. Rogers

**Affiliations:** Addison


### ART. VII.—*Case of Purpura Hæmorrhagica.*

ADDISON, June 1, 1849.

Dr. FLINT—*Dear Sir:*—Permit me to place at your disposal an account of a novel case, to me at least, being unlike any thing that I have yet seen in books, or have described by teachers, or met with in practice. I am therefore unable to give it a name; the case as extracted from my note book is as follows:—*

* NOTE.—The name is prefixed by Editor.

*April* 4, D. G.—A lad aged 18 months has been sick some ten days; upon his first attack a neighboring physician was called, and diagnosed it inflammation of the bowels; for the last two or three days has had hemorrhage pr. anus, urethra, mouth, nose, and, its parents said, from his eyes; very pale, pulse small and feeble, owing to the timidity of the child was unable to count it, in short, the appearance indicated general debility. The child, before this attack, was uncommonly healthy.

Prescribed.—Acetate lead, quinine, carbonate iron, six grs. in solution, making two ounces, one teaspoonful once in two hours.

*April* 6.—Hæmorrhage ceased, appetite improved—discontinued the lead, but continued the iron and quinine.

*April* 8.—No material change in appearance, cuticle nearly transparent, upon the forehead was seen two or three spots of the size of a small fluid-drop of blood, and as red, beneath the cuticle; recommended nutritious diet, milk, broths, &c., continued tonics.

*April* 10.—Loss of appetite, frequent stools and dark color, feeble pulse, surface cool; these red spots increased in number and minuteness, many not larger than the head of a pin, but a few upon the face and upper extremeties, more on the thighs, and still more upon the abdomen; these spots would not disappear by pressure, there was no elevation of the surface, epidermis being smooth and transparent, the blood having exuded from the vessels, and appearing to have extravasated, a portion of the cinutis vera.

*April* 15.—No material change, the patient seeming to be failing gradually, pulse small and feeble, tonics continued, with nutritious diet.

*April* 21—4 P. M.—Patient continued to fail, circulation weak, spots upon the upper extremeties were still isolated, upon the thighs and abdomen had united, and more purple; at 11 P. M. the patient expired, these spots having become nearly "black." No post mortem was held.

Yours, &c.,

S. C. ROGERS, M. D.

